# Treatment pathways of Japanese prostate cancer patients - A retrospective transition analysis with administrative data

**DOI:** 10.1371/journal.pone.0195789

**Published:** 2018-04-25

**Authors:** Stephane Cheung, Yukinobu Hamuro, Jörg Mahlich, Masahiko Nakayama, Akiko Tsubota

**Affiliations:** 1 Kwansei Gakuin University, Nishinomiya, Japan; 2 Janssen KK, Health Economics, Tokyo, Japan; 3 DICE, University of Dusseldorf, Dusseldorf, Germany; 4 Janssen KK, Medical Affairs, Tokyo, Japan; Texas Technical University Health Sciences Center, UNITED STATES

## Abstract

**Background:**

Limited availability of real-world data that describe treatment patterns of Japanese prostate cancer (PCA) patients.

**Methods:**

A biweekly transition analysis of PCA treatment was performed for patients with PCA diagnosis and a specific treatment between 2010 and 2015. To account for different cancer stages, two patient populations were analyzed. The first group consisted of patients on medication for hormone-sensitive prostate cancer (HSPC). The second group is comprised of patients who ended up receiving specific therapy for castration-resistant prostate cancer (CRPC). For each treatment, the average of treatment duration and the portion of patients transitioning to a consecutive treatment was calculated.

**Results:**

We identified 59,626 patients from the Japanese administrative database with a PCA diagnosis and specific treatment. In the first year of our observational study 786 patients commenced a HSPC treatment and 695 received a CRPC specific therapy Among the HSPC group, we found that combination hormonal therapy, comprised of a gonadotrophin releasing hormone agonist or antagonist with an antiandrogen was more common than monotherapy. The results of the CRPC group indicated that chemotherapy administration was for a shorter time period in a real-world setting as compared to published clinical studies.

**Conclusion:**

Utilizing a novel method to visualize real-world treatment pathways for PCA patients we found that real treatment pathways are in line with international guidelines.

## Introduction

Historically age-standardized mortality rates for prostate cancer (PCA) have been lower in Japan compared to the Western countries. However, the incidence in Japan has been gradually rising and diminishing the gap with other developed countries [1]. Nevertheless, a recent analysis reported lower mortality from PCA in Japan compared to countries such as Australia or New Zealand [2]. In line with increasing prevalence and mortality, the economic burden of PCA in Japan has also steadily increased and expected to rise further in the future. The estimated costs of PCA in Japan was between 354.7–378.3 billion Yen in 2014 with projections to reach 385.3–474.1 billion Yen in 2020 which translates into 3.76–4.6 billion USD. Direct costs associated with the inpatient and outpatient treatments, laboratory and diagnostic tests, as well as pharmaceuticals accounted for 60–75% of the total overall costs [3]. In particular, the later stage treatment of PCA is more expensive. A recent claims database analysis revealed health care costs of approximately 97,506 Yen (952 USD) per patient per month for castration resistant prostate cancer (CRPC) outpatients after docetaxel treatment. Hospitalization (inpatient care) add 333,350 Yen (3,790 USD) per month while other chemotherapy treatments elevated costs by 126,647 Yen (1,236 USD) per month [4].

Limited data is available on treatment of PCA patients in a real-world setting. A retrospective chart review of patients with metastatic castration resistant prostate cancer (mCRPC) (N = 445) concluded that 43.64% of patients during first line and 40.68% fourth line treatment received androgen deprivation therapy (ADT). Administration of enzalutamide and abiraterone were also common practices, though more so in later stages due to relatively recent accessibility to these drugs (used among 14.46% and 8.73%, of patients in 1st line and 40.68% and 20.34%, respectively, in 4th line) [5]. Therefore, we sought to study the treatment path for Japanese PCA patients from a considerably large administrative data set utilizing a state transition diagram approach. This strategy has been used to describe biological networks [6], yet the application is reasonably new in health services research.

## Methods

### Study population

We utilized commercially available hospital claims data from Medical Data Vision Co., Ltd (MDV). This is an administrative database for all inpatients and outpatients in Japan and includes approximately 4,400,000 patients. This number represents about 3% of the total Japanese population. The age distribution in the database is 13.5% for 0–14 years old, 52.4% for 15–64 years old, and 34.1% for 65 years old closely representing that of the general population [7]. The data were extracted from hospital electronic information systems derived from 147 acute-phase hospitals throughout Japan. The hospitals operate 40,000 beds and are registered as Diagnosis Procedure Combination (DPC) organizations. The DPC is a Diagnosis-related group (DRG)-like flat fee system that was introduced in 2003 for large hospitals in Japan [8]. The MDV database has been accessed to analyze a wide range of therapeutic areas in Japan such as rheumatoid arthritis [9,10], influenza [11], or schizophrenia [12].

As the data from Medical Data Vision Co., Ltd (MDV) had been anonymized, the Ethical Guidelines for Epidemiological Research (Ministry of Education, Culture, Sports, Science and Technology, and Ministry of Health, Labour and Welfare of Japan), which require ethics approval and informed consent, are not applicable to this study.

Selection criterion was a confirmed PCA diagnosis (International Statistical Classification of Diseases and Related Health Problems 10th version (ICD10) codes C61) and a PCA specific medication. The drugs used in this study to define PCA-related treatments are reported in Table 1.

**Table 1:** Drugs list for patient selection.

Patient groups	Drug name	Drug class	Abbreviations	Drug dosage, duration & route of administration
CRPC	Cabazitaxel	Microtubule inhibitor	Cab	42mg/m2, 3weeks ^1^
	Enzalutamide	Androgen Receptor Antagonists	Enz	oral drug
	Abirateron	Cyp17A1-Inhibitor	Abi	oral drug
	Estramustine	Antimicrotubule agent	Estra	oral drug
	Docetaxel	Microtubule inhibitor	Doc	100mg*, 3weeks ^2^
HSPC	Bicalutamide	Antiandrogen	Bic	oral drug
	Flutamide	Antiandrogen	Flu	oral drug
	Ethinyl estradiol	Estrogen	Est	oral drug
	Goserelin acetate	Gonadotrophin‐releasing hormone agonist	GnRHa	3.6mg/4weeks or 10.8mg/12-13weeks^3^
	Leuprorelin acetate	Gonadotrophin‐releasing hormone agonist		11.25mg/12weeks, 3.75mg/4weeks^4^
	Degarelix acetate	Gonadotropin-releasing hormone antagonist		1st time 120mg/part and from 2nd time 80mg/4 weeks ^5^

* 100mg = 60mg*1.695m2 (1.695m2 is calculated from 60kg weight and 170cm height)

^1^ Package insert of Cabazitaxel, http://www.pmda.go.jp/PmdaSearch/iyakuDetail/780069_4240410A1020_1_04#118

2 Package insert of Docetaxel http://www.pmda.go.jp/PmdaSearch/iyakuDetail/780069_4240405A1037_1_07#183

3 Package inserts of Goserelin acetate, http://www.pmda.go.jp/PmdaSearch/iyakuDetail/670227_2499406G1025_1_20#92, http://www.pmda.go.jp/PmdaSearch/iyakuDetail/670227_2499406G3028_1_15#93

4 Package inserts of Leuprorelin acetate, http://www.pmda.go.jp/PmdaSearch/iyakuDetail/400256_2499407G3030_1_04#102

http://www.pmda.go.jp/PmdaSearch/iyakuDetail/400256_2499407D1031_1_04#300

5 Package inserts of Degarelix acetate, http://www.pmda.go.jp/PmdaSearch/iyakuDetail/800126_2499412D1024_1_05#116

Study eligibility period was from July 2010 to June 2015 inclusive. Patients identified with loss of data were removed. A data loss can occur when a certain hospital does not make available the data from the hospital to the data base provider. Because this is an administrative dataset, information on the cancer stage was unavailable. In order to reflect different treatment patterns by disease severity we divided the population into two subpopulations that are reasonably similar in terms of disease severity.

To include a patient population that is sufficiently homogeneous we defined two different patient cohorts that were apparently in different disease stages:

The hormone sensitive prostate cancer (HSPC) group included PCA patients undergoing hormone type treatments with the following characteristics:

□ Commence hormone therapy for PCA as defined in Table 1 between July 2010 and June 2011
□ No other cancer diagnosis was made before June 2010.
□ No prescriptions of drugs related to PCA was received according to Table 1 from January 2010 to June 2010 (washout period).
□ No surgical or radiation intervention between July 2010 and June2011.

This cohort represented cancer patients in an intermediate stage, typically after a recurrence following surgery or radiation intervention. Some of the patients deemed ineligible for surgery or radiation therapy were directly commenced on hormone therapy. Follow-up period of this cohort was 5 years.

The second group consisted of CRPC patients eventually receiving CRPC type treatments with the following characteristics:

□ Undergone CRPC drug treatment as defined in Table 1 between July 2014 and June 2015.

The CRPC cohort represented late-stage PCA patients. We have observation period of up to five years for this group.

### Transition analysis

**Fig 1:**
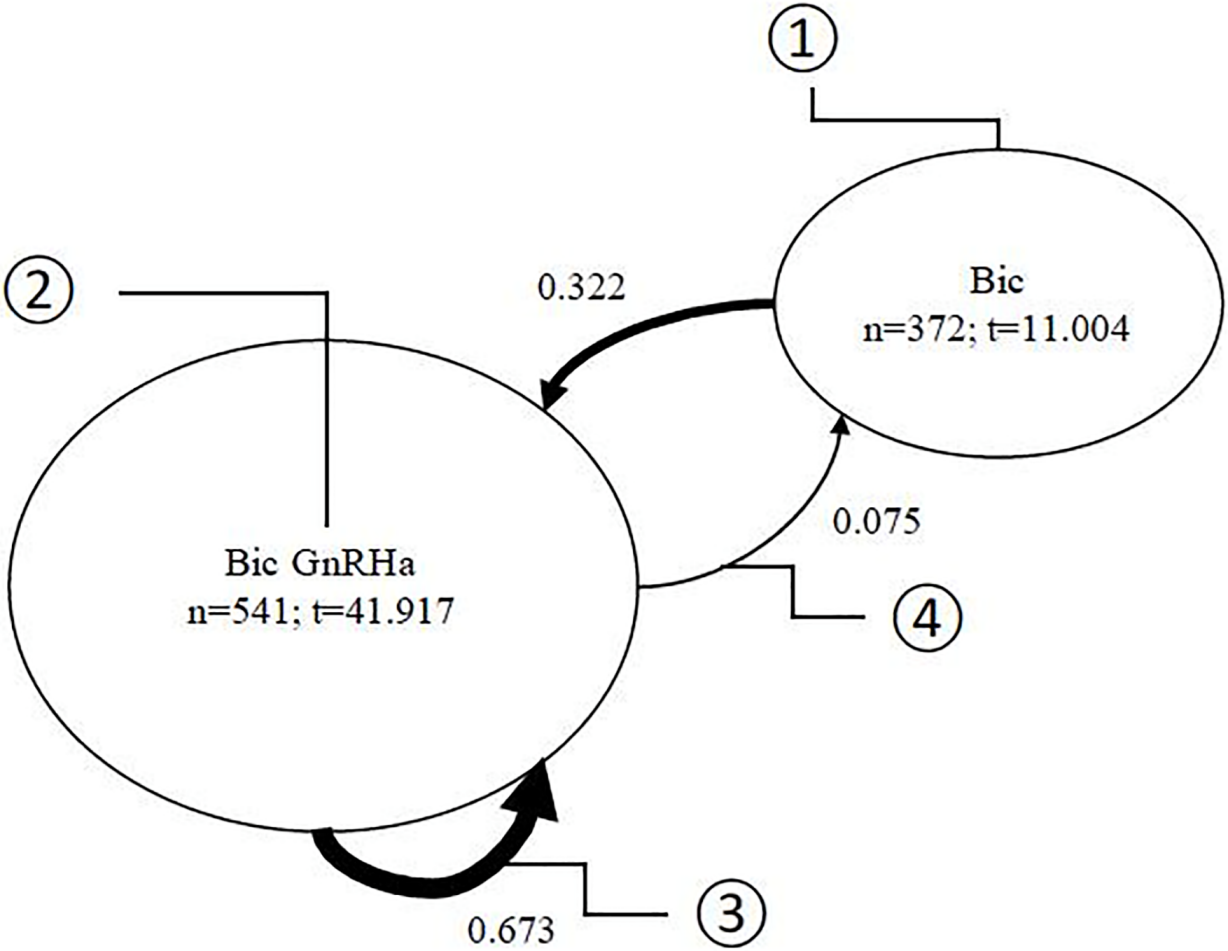
Edges and nodes of a transition diagram.

Transition Analysis is a common method applied in computer science [13]. A transition diagram is a graphical representation of sequence of PCA treatment patterns that transfers from initial treatment through intermediate treatments (refer to Fig 1 for an example of a transition diagram). So-called nodes (①) represent treatments which are characterized by a specific treatment or by a combination of treatments. In our example ① represents a hormone treatment with bicalutamide, while treatment ② is a combination treatment of bicalutamide together with either one of treatments in the gonadotrophin releasing hormone agonists and antagonist (GnRHa) group including goserelin acetate, leuprorelin acetate or degarelix acetate. These report the number of patients that had been in this treatment state during our observation period. Double counting when the same treatment changes reoccur for the patient at a latter period was not permitted. The nodes report the average time (in weeks) a patient was in the respective treatment state.

The edges describe transitions from one treatment to the next. Edges can be recursive such as in ③ if the treatment continues in the following time period. An edge is called directed if the transition is towards a different treatment (as in ④). The thickness of the edge arrow reflects the ratio of patients in the population group who go through this edge to all patients. The thicker the edge, the more popular is this treatment path. The probability of switching treatment after a time unit is depicted next to the edges. This value provides the share of patients that go to the respective next treatment. Instead of probabilities, we also report the absolute number of patients next to the arrows.

In our analysis, we used biweekly time intervals. Also, for a better representation of the data, we defined the threshold for the number of transitions to be 15 patients. That is so that we do not draw an edge if less than 15 patients move from one treatment to another treatment within the observation period.

In order to accurately reflect the length of different medication days’ supply in the transition diagram, the following rules for different route of administration of a drug were followed in order to define actual days’ supply:

1. Oral drugs: The number of supply days is a variable that is available in the data set.
2. Injection drugs: The number of supply days is calculated by imposing a recommended dosage based on the package insert. The actual prescribed dosage is available from the data set.

For oral drugs, if a patient has a refill for the prescription while still on supply of the old prescription, this surplus supply is carried over to the next prescription refill. For Injection, there is no carry over, and the medication days are reset upon every injection.

## Results

### Patient population

**Fig 2:**
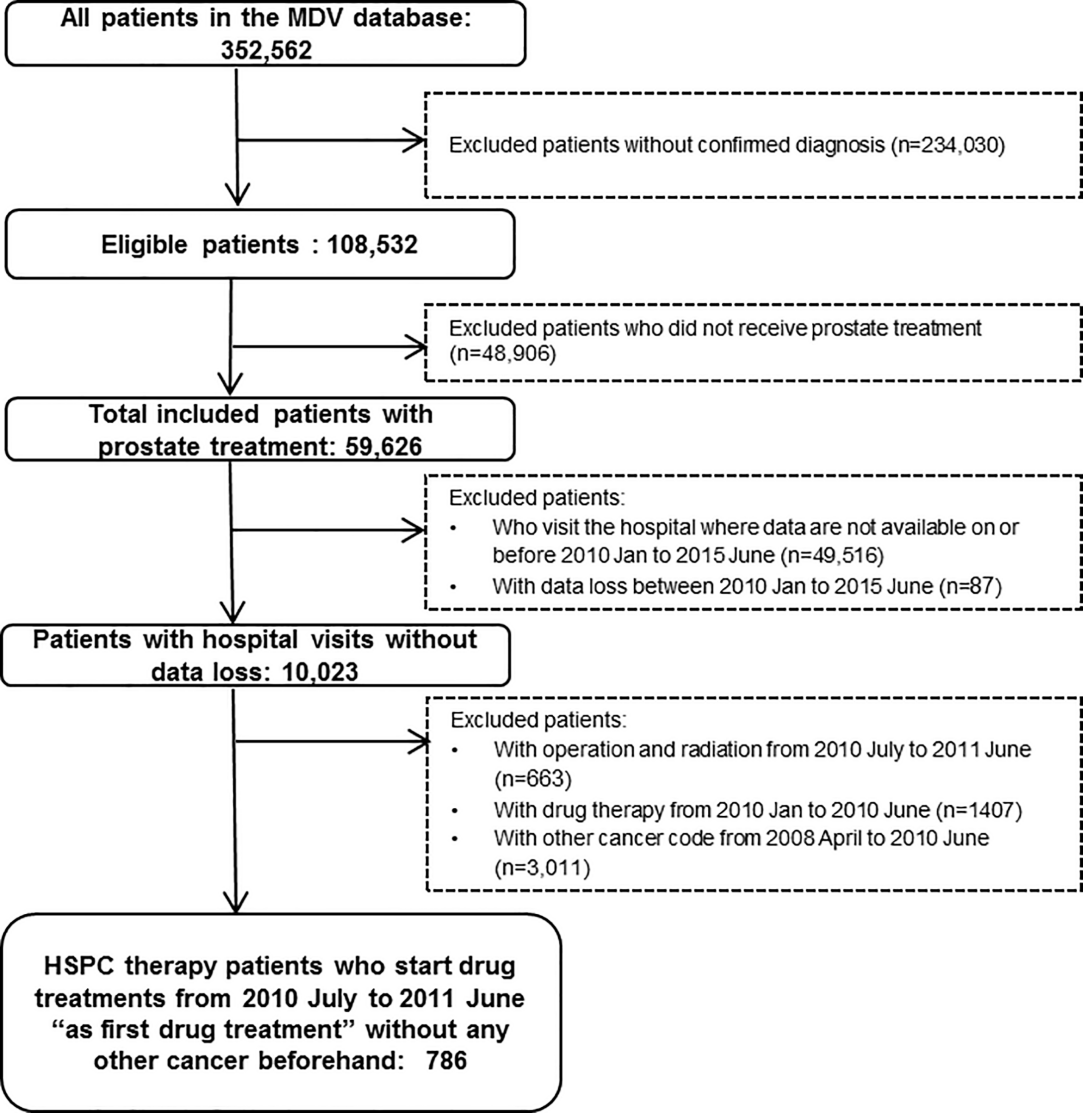
Patient selection and composition of the HSPC cohort.

**Fig 3:**
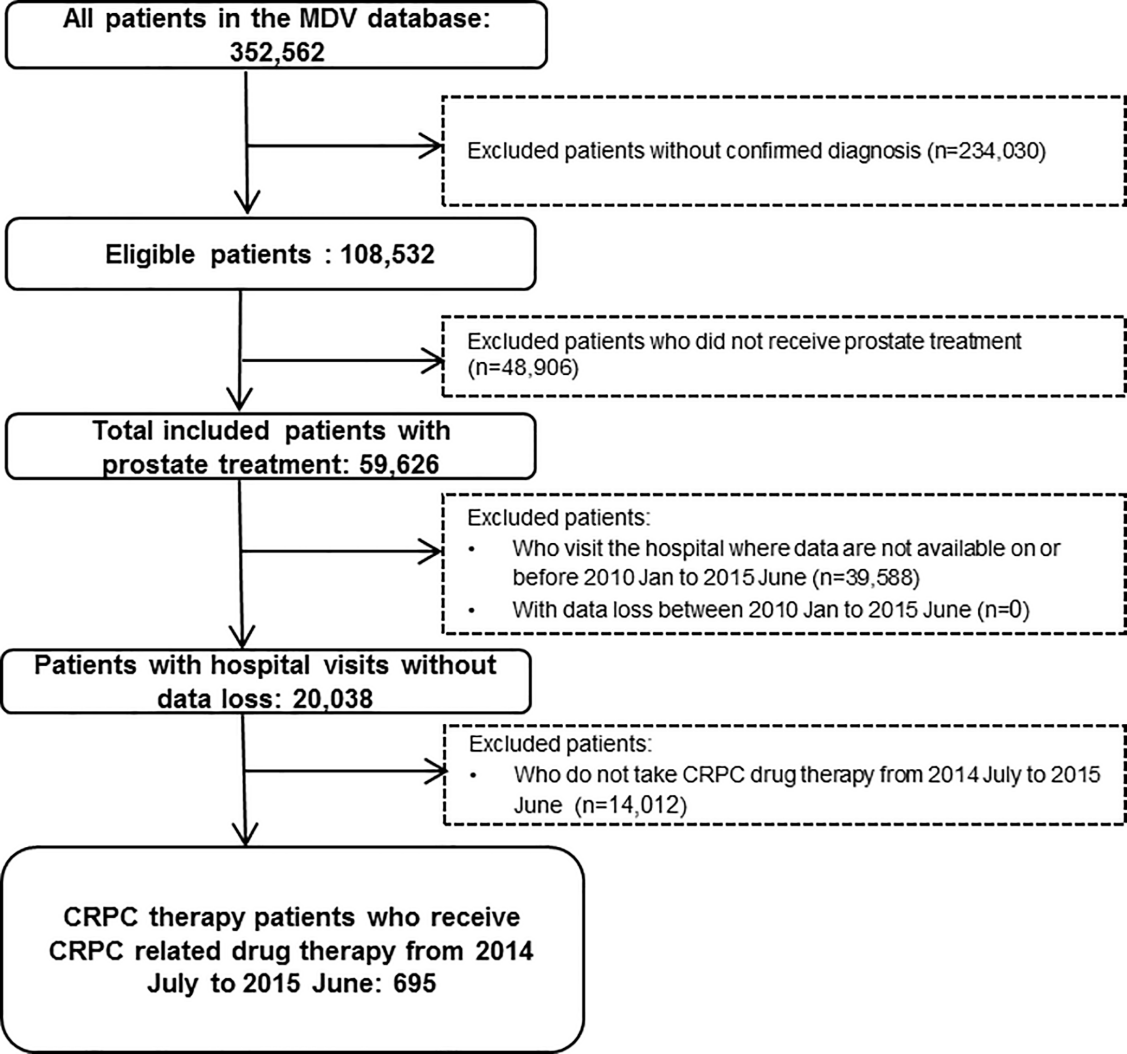
Patient selection and composition of the CRPC cohort.

We identified 352,562 patients with a confirmed ICD10 codes C61 in the database, from which 59,626 were under medical treatment, with 786 already commenced a HSPC treatment and 695 a CRPC specific therapy. The course of patient selection and the composition of the two cohorts are presented in Figs 2 and 3.

Table 2 reports the characteristics of both the overall population and the two subpopulations.

**Table 2:** Descriptive Statistics of patient population.

Population from July 2010 to June 2015		Number of patients receiving prostate cancer treatment	HSPC group	CRPC group	
Overall	Total number	59,626	786	695	
	Mean Age	77.5	78.27	76.26	
			p < 0.000 (t-test)		
	Median Age	78	79	77	
			p < 0.000 (Wilcoxon test)		
	SD	8.23	8.11	7.8	
Age^1^	<60 count (ratio)	981 (1.6%)	8 (1.0%)	14 (2.0%)	
	60–69 count (ratio)	9,426 (15.8%)	107 (13.6%)	124 (17.8%)	
	70–79 count (ratio)	23,789 (39.9%)	313 (39.8%)	315 (45.3%)	
	≥ 80 count (ratio)	25,430 (42.6%)	358 (45.5%)	242 (34.8%)	
Length of treatment [in weeks] ^2^	Mean	72.08	110.13	187.16	
	Median	49.14	90	184.29	
% late stage treatment (CRPC)^3^	Count (ratio)	4,853 (8.1%)	83 (10.6%)	695 (100%)	
Number of deaths in hospital	Count (ratio)		51 (6.49%)	50 (7.19%)
Number of patient deaths in hospital per week	Ratio		0.059%	0.038%	

^1^Age as of 2015 June

^2^ Length of treatment from initial prostate treatment to last one in act data

^3^ Late stage treatments include drugs used for CRPC patient group.

While there are marginal differences in the demographic factors of both patient populations, the CRPC group was treated for approximately twice as long as the HSPC group. Of the HSPC group 81% have bone metastases codes with 11% transferring to CRPC treatment within 5 years. The total number of deaths in hospital both as crude numbers as well as adjusted per patient week are represented in Table 2. The adjusted mortality rate among the CRPC patients is lower compared to the HSPC patients. S1 Table reports the top five treatment combinations for each patient groups.

Regarding comorbidities that are reported in Table 3, CRPC patients have more comorbidities than the HSPC patient group. This is true for both PCA related comorbidities such as bone metastatic, as well as for other comorbidities such as diabetes.

**Table 3:** Most common comorbidities.

Disease Name (Disease Code)	Overall population (%)	HSPC (%)	CRPC (%)	P value
N	59,626	786	695	
Bone metastatic cancer (1985007)	6,966 (11.7%)	98 (12.5%)	169 (24.3%)	P<0.000
Prostate cancer bone metastasis (8842788)	8,530 (14.3%)	92 (11.7%)	215 (30.9%)	P<0.000
Enlargement of prostate (8836591)	33,786 (56.7%)	341 (43.4%)	392 (56.4%)	P<0.000
Osteoporosis (7330006)	5,965 (10.0%)	49 (6.2%)	123 (17.7%)	P<0.000
Diabetes (8 disease codes) ^1^	36,012 (49.1%)	280 (35.6%)	398 (57.3%)	P<0.000
Liver dysfunction (24 disease codes) ^2^	22,895 (31.2%)	213 (27.1%)	415 (59.7%)	P<0.000
Gastric cancer (1519006)	6,370 (10.7%)	66 (8.4%)	109 (15.7%)	P<0.000
Colorectal cancer (1539004)	6,840 (11.5%)	89 (11.3%)	109 (15.7%)	0.01712
Pancreatic cancer (1579002)	3,208 (5.4%)	46 (5.9%)	71 (10.2%)	0.00261
Lung cancer (1629006)	5,465 (9.2%)	65 (8.3%)	110 (15.8%)	P<0.000
Bladder cancer (1889005)	7,618 (12.8%)	65 (8.3%)	113 (16.3%)	P<0.000
Lymph node metastasis (1969001)	4,008 (6.7%)	48 (6.1%)	115 (16.5%)	P<0.000
Metastatic lung cancer (1970005)	5,235 (8.8%)	25 (3.2%)	140 (20.1%)	P<0.000

^1^ 250001 Insulin resistant diabetes, 2500013 Diabetes, 2500015 Type 2 diabetes, 2500031 borderline diabetes, 2509003 Steroid diabetes, 8843389 Steroid diabetes with keto acidosis, 8843395 Steroid diabetes without complications, 8843439 Diabetes without complications

^2^ 701005 Acute hepatitis, 2390027 Liver tumor, 5714005 Chronic hepatitis, 5715012 Decompensated cirrhosis, 5722003 hepatic encephalopathy, 5733005 hepatitis, 5733014 drug-induced hepatitis, 5738002 Hepatic dysfunction, 5738014 Hepatic failure, 5739013 Drug-induced hepatitis failure, 5739014 Hepatic disorder, 5758029 Intrahepatic cholestasis, 7823029 Hepatic edema, 7891001 Hepatomegalia, 7948001 Abnormal liver function test, 8830386 Allergic hepatitis disease, 8831511 Hepatic sclerosis, 8831512 Cirrhosis hepatitis, 8831536 Hepatopathy, 8831588 Hepatorenal syndrome, 8831607 Hepatic fibrosis, 8832315 Acute hepatic failure, 8840321 Chronic hepatic failure, 8845885 Acute drug-induced hepatitis

### Transition analysis

**Fig 4:**
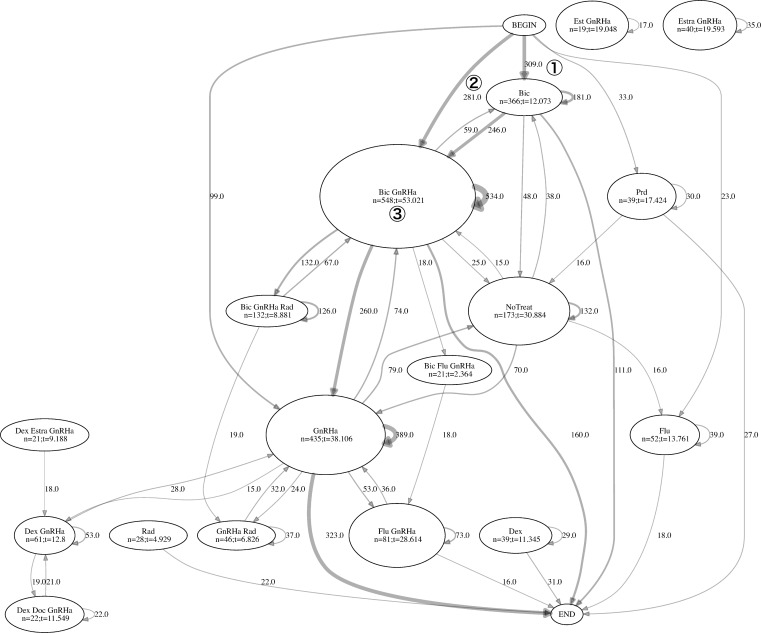
HSPC transition chart. Pre: prednisolone, Dex: dexamethasone, Notreat: no treatment, i.e. a period of 2 weeks without medication supply.

Transition charts are presented in Fig 4 for HSPC population.

As there are a high number of transitions between treatments with low frequency among patients in the HSPC population, a transition diagram with the minimum edge frequency of 15 is selected for ease of understanding. The transition analysis revealed 309 patients (① in the chart, 309/786 = 39.3% of the HSPC) commenced their treatment with the anti-androgen bicalmid (mean treatment length of 12.073 weeks) and 281 patients (②, 35.8%) of the HSPC patients with a combination of bicalmid and GnRHa type, which is referred to as “combined androgen blockade” (CAB). Treatment length for CAB patients was 53.021 weeks for 548 patients (③, 69.7) on this therapy.

**Fig 5:**
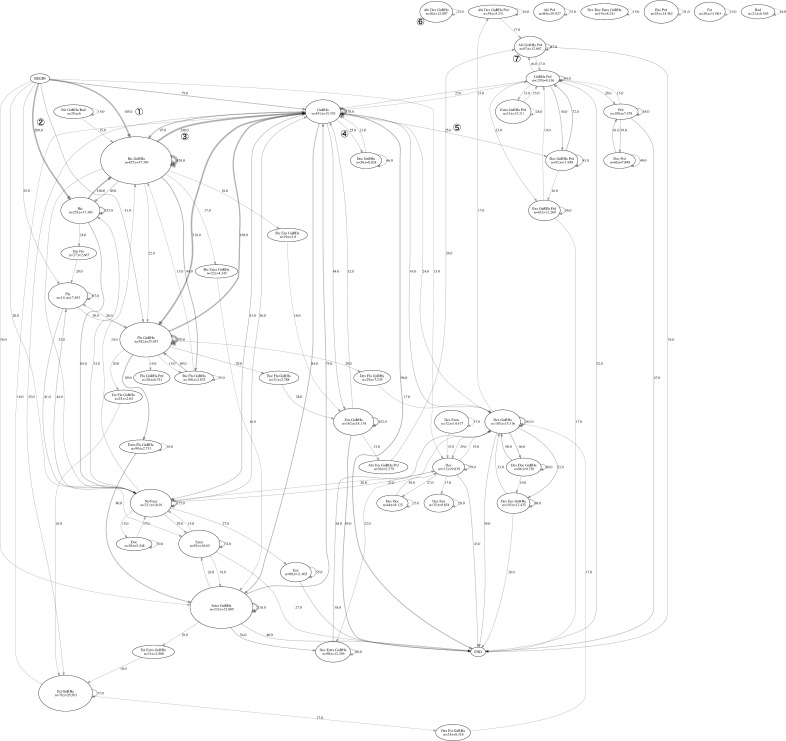
CRPC transition chart. Notreat: no treatment, i.e. a period of 2 weeks without medication supply.

The CRPC patient group transition analysis is reported in Fig 5. Treatment paths for the CRPC group were more complex and individualized compared with the HSPC group. Initially, 185 CRPC patients (①, 26.6%) were on CAB therapy and 209 (②, 30.1%) on bicalmid monotherapy. The length of CAB therapy was 37.393 weeks.

240 of patients (③, 34.5%) were changed to GnRHa therapy thereafter. Then, patients are observed progressing to docetaxel-based chemotherapy, including 25 patients (④, 3.6%) transitioned to docetaxel and GnRHa therapy for 8.024 weeks, while 25 other patients (⑤, 3.6%) proceeded to docetaxel, GnRHa, and prednisone combined therapy for 11.959 weeks.

The transition chart also shows 26 patients (⑥, 3.7%) having a combined treatment of abiraterone acetate, dexamethasone, and GnRHa for an average length of 12.897 weeks, while 97 patients (⑦, 14.0%) have a combined treatment of abiraterone acetate, GnRHa, and prednisone for an average length of 12.667 weeks as well. Moreover, enzalutamide was found to be frequently administered with either types of steroids.

## Discussion

### HSPC group

Since its introduction about 70 years ago by Huggins and Hodges [14], hormonal therapy has played a key role in treatment of advanced PCA also in Japan. Hormone therapy after surgical castration or in patients not eligible for surgical castration in Japan is a very common practice. Data from a Japanese PCA registry reported 45% of localized PCA patients were treated by hormonal therapy [15]. Contrary to Western countries, in Japan such therapy is widely practiced in early stages of the disease including non-metastatic localized cancer and invasive localized cancer [16]. Moreover, Japanese are reportedly more respondent to hormone therapy than Caucasian men [17]. Adverse events also seem to occur less frequently in the Japanese population [18]. Despite its common practice hormone therapy is not without adverse drug reaction (ADR) risks such as vasomotor flushing, loss of libido, erectile dysfunction, cognitive decline, arterial stiffness, anemia, fatigue, gynecomastia, mastodynia, osteoporosis leading to fractures, obesity, sarcopenia, and cardiovascular disease [19].

One concerning issue that has been extensively discussed in literature is whether monotherapy or CAB should be applied. The latter treatment strategy is defined by concomitant use of a GnRHa together with an antiandrogen therapy. GnRHa block the production of testosterone from the brain while the antiandrogen competitively inhibit androgen binding to androgen receptors at the PCA cellular level. This should render hormone therapy more effective. Previously, a large controlled trial confirmed association of the CAB (in this case leuprolide combined with flutamide) with a 25% survival advantage over monotherapy (leuprolide alone) [20]. Overall, a meta-analysis indicated a slight but statistically significant survival benefit with combination hormonal therapy using nonsteroidal antiandrogens [21]. However, the question is whether such minor benefits outweigh the increased risk of ADRs and additional accumulated costs. Individual studies have reported differences between antiandrogens in terms of both tolerability and efficacy; for example, bicalutamide has been shown to be better tolerated than flutamide, and may be associated with improved survival [22]. On the other hand, an Italian study did not find differences in health-related quality of life between different hormones [23]. The ADRs of combination therapy are also worth to mention here as antiandrogen monotherapy is less likely to induce muscle loss and reduce libido than the GnRHa agonists. For example, only about 59% in the bicalutamide monotherapy arm lost their libido compared to about 80% of men 85% in the CAB group [24]. Furthermore, a lower risk of developing osteoporosis is associated with monotherapy [25]. Consequently, international guidelines are more skeptical about CAB therapy. The European Association of Urology guideline for instance states that “it remains debatable whether this small advantage can be meaningful when applied to everyday clinical practice” [26]. In the Japanese Clinical Practice Guideline for Prostate Cancer 2012, CAB is still recommended without restrictions [27]. The present study demonstrated continued wide use of CAB in the treatment of PCA in Japan.

Another issue with regards to hormonal treatment is whether it can be discontinued for a treatment break (intermittent androgen deprivation (IAD)) or if it is more beneficial to continue treatment as long as possible (continuous androgen deprivation (CAD)). The rationale for a treatment break is that patients are allowed to restore quality of life as before such as returning libido and sexual health. Moreover, such a pause in treatment may potentially delay hormone resistance that eventually develops in men under long-term hormone therapy. However, there is some evidence to recommend use of IAD instead of CAD for the treatment of men with relapsing, locally advanced, or metastatic PCA who achieve a good initial response to androgen deprivation [28]. For Japan, our transition analysis suggests that hormone therapy is often interrupted for a treatment break (IAD).

### CRPC group

In the CRPC group, treatment was found to be more tailored for the individual patient with no single treatment path option observed for the majority of patients. The recommended treatment process for the late-stage cohort is described as follows [29].

### Treatment options after ADT and before chemotherapy

Abiraterone acetate in combination with prednisone or prednisolone or enzalutamide is generally recommended as a standard option for treating mCRPC in individuals with mild or no symptoms after ADT has been unsuccessful, and before chemotherapy is indicated. This treatment pattern was observed in the MDV data for Japan. We have not detected a pathway from enzalutamide to chemotherapy due to relatively recent introduction of enzalutamide in May 2014 with a post-chemotherapy indication that had been expanded to pre-chemotherapy in Oct 2015, after our study observation period.

### Chemotherapy

For chemotherapy-naive CRPC, the standard care of chemotherapy commenced in 2004 when docetaxel plus prednisone showed a significant improving of survival compared to the mitoxantrone plus prednisone [30].

According to the recommendations of National Institute for Health and Care Excellence (NICE) guidelines, treatment with docetaxel should be stopped at the completion of planned treatment of up to 10 cycles [31]. Since docetaxel should be injected once every 3 weeks, 10 cycles would correspond to a treatment duration of 30 weeks. Our transition analysis, however, revealed that docetaxel was administered approximately 8 to 11 weeks in real world clinical practice. This might be due to the concern for severe ADRs associated with chemotherapy and hence Japanese physicians are very cautious in their treatment approach. In addition, the NICE guideline does not recommend repeated cycles of treatment with docetaxel if the disease recurs after completion of the planned course of chemotherapy. Our findings were consistent with this recommendation.

### Treatment options after chemotherapy

For patients with progression after docetaxel, there are limited treatment options that have been shown to prolong survival. Cabazitaxel is a second-line chemotherapy and recommend to docetaxel failures. Using an edge support of 15 patients, we could not find cabazitaxel usage. This was possibly due the relatively recent launch of cabazitaxel in July 2014. For this reason, the number of patients undergoing treatment is still too small to reach definitive conclusions.

In conclusion, we have observed treatment pathways that are very much unique to the individual and probably contingent on medical parameters that are unobserved in our database. The variability of treatment options in this indication and progressive innovations sometimes poses as a significant challenge to reach treatment decisions purely based on clinical evidence alone, since not all possible combinations have been compared against each other. The complexity of treatment options further calls for a strong involvement of patients in treatment decision making [32].

### Limitations

One of the limitations of this study was that claims data analysis in general can utilize only a very limited set of medical parameters, which are essential in determining the treatment flow of patients in oncology. This is an inherent limitation of claims database analysis in general. Furthermore, the MDV database is generated in large Japanese DPC hospitals, which does not necessarily reflect general treatment patterns in Japan. The CRPC Group is possibly more representative of the overall Japanese population because later stage cancers are unlikely to be treated in small private practice clinics as most are not covered by the national database. On the other hand, early stage PCA and hormone therapy can be treated in smaller clinics as for this particular subgroup we cannot rule out the possibility of a bias toward patients with more severe presentation of symptoms compared to the general patient population.

## Conclusion

Utilizing a novel method to visualize real-world treatment pathways for PCA patients, we found that real treatment flows are considerably in line with clinical guidelines. We recommend this novel methodology to be adopted in other indications as well to study and analyze real-world treatment pathways in accordance with clinical evidence.

## Supporting information

S1 Table Most common treatment combinations. (DOCX)
