# Supplementary material for: Treatment pathways of Japanese prostate cancer patients - A retrospective transition analysis with administrative data
Source: PLoS One. 2018 Apr 25;13(4):e0195789. doi: 10.1371/journal.pone.0195789 (PMC5919000; doi:10.1371/journal.pone.0195789)
Supplement: S1 Table — (DOCX) [file pone.0195789.s001.docx]

**Supplementary Table 1: Most common treatment combinations**

| HSPC |  | CRPC |  |
| --- | --- | --- | --- |
| Bic GnRHa | 68.6% | GnRHa | 64.9% |
| GnRHa | 54.4% | Bic GnRHa | 61.4% |
| Bic | 45.8% | Flu GnRHa | 55.0% |
| Bic GnRHa Rad | 16.5% | Bic | 36.7% |
| FluGnRH | 10.1% | Estra GnRHa | 33.5% |

Bic: Bicalutamide, GnRHa: hormone antagonist, Flu: Chlormadinone acetate Flutamide, Rad: Radiation, Estra: Antimicrotubule agent.
